# Draft Genome Sequence of *Arthrobacter* sp. Strain UCD-GKA (Phylum *Actinobacteria*)

**DOI:** 10.1128/genomeA.01599-16

**Published:** 2017-02-09

**Authors:** Gregory N. Kincheloe, Jonathan A. Eisen, David A. Coil

**Affiliations:** a University of California, Davis Genome Center, Davis, California, USA; b Department of Evolution and Ecology and Department of Medical Microbiology and Immunology, University of California Davis, Davis, California, USA

## Abstract

Here we present the draft genome of *Arthrobacter* sp. strain UCD-GKA. The assembly contains 4,930,274 bp in 33 contigs. This strain was isolated from the handle of a weight bar in the UC Davis Activities and Recreation Center.

## GENOME ANNOUNCEMENT

Members of the genus *Arthrobacter* are aerobic, Gram-positive bacteria (1) primarily known for switching between the bacilli and cocci shapes depending on the environmental conditions (2). They are commonly found in soil.

*Arthrobacter* sp. strain UCD-GKA was isolated from a squatting bar located in the weight room of the UC Davis Activities and Recreation Center. This was part of an undergraduate research project to provide microbial reference genomes of bacteria isolated from the built environment (http://www.microbe.net). A sterile cotton-tipped swab was used to rub the handles of the squat bar and then applied to a lysogeny broth (LB) agar plate. The plate was incubated at 37°C for 4 days. A single colony was dilution streaked, and once isolated, was used to make an overnight culture that was also incubated at 37°C. DNA extraction from this overnight culture utilized the Promega Wizard genomic DNA purification kit. The 16S rRNA gene was amplified using PCR with the 27F and 1391R primers. The PCR product was then purified and used for Sanger sequencing. The resulting consensus sequence was analyzed using NCBI BLAST (3) and phylogenetic tree building using an alignment of related sequences from the Ribosomal Database Project (RDP) (4). A maximum-likelihood phylogenetic 16S rRNA gene tree was inferred using Fast Tree (5), and was visualized in Dendroscope (6). This preliminary tree included other *Arthrobacter* species, including *A. antarcticus*, *A. psychrophenolicus*, *A. gangotriensis*, and *A. sulfureus*.

For whole-genome sequencing, a paired-end library was prepared using a KAPA HyperPlus library prep kit (KAPA Biosystems). A portion of an Illumina MiSeq sequencing run generated 383,256 paired-end reads with a read length of 300 bp. After quality trimming and error correction were completed by the A5-miseq assembly pipeline (7, 8), 356,510 quality reads remained in 33 contigs, with 15× coverage and a GC content of 65.7%. Genome completeness was estimated using the Phylosift software (9), which uses a reference list of 37 highly conserved, single copy marker genes (10), 36 of which were found in the assembly in a single copy. The missing marker, RNase H11, was found in 98.5% of bacteria surveyed when these markers were compiled and so it is unclear whether this marker is genuinely absent or the assembly is incomplete. With the use of another metric, which was provided by CheckM (11), completeness was estimated at 99.5% complete.

Annotation was performed using RAST (12). *Arthrobacter* sp. strain UCD-GKA is predicted to contain 4,584 coding sequences and 70 noncoding RNAs. An almost full-length 16S rRNA gene sequence of 1,450 bp was obtained and analyzed as described above. Within the resulting tree, *Arthrobacter* sp. strain UCD-GKA was found in a clade containing mostly *Arthrobacter sulfureus*. However, the tree contained many polyphyletic groups that did not correspond with the 16S rRNA gene phylogeny and therefore we did not assign a species name to this isolate (https://figshare.com/articles/Arthrobacter_Tree/4234637).

### Accession number(s).

This whole-genome shotgun project has been deposited at DDBJ/EMBL/GenBank under the accession number MOLR00000000. The version described in this paper is version MOLR01000000.
